# Are serum levels of inflammatory markers associated with the severity of symptoms of bipolar disorder?

**DOI:** 10.3389/fpsyt.2022.1063479

**Published:** 2023-01-20

**Authors:** Xiuhua Wu, Zhongcheng Chen, Yingtao Liao, Zhihua Yang, Xiaolin Liang, Nianhong Guan, Zhaoyu Gan

**Affiliations:** ^1^Department of Psychiatry, The Third Affiliated Hospital of Sun Yat-sen University, Guangzhou, Guangdong, China; ^2^Department of Laboratory Medicine, The Third Affiliated Hospital of Sun Yat-sen University, Guangzhou, Guangdong, China

**Keywords:** bipolar disorder, symptomatic severity, inflammatory markers, stage, pathophysiology

## Abstract

**Background:**

To explore the relationship between serum levels of inflammatory markers and symptomatic severity of bipolar disorder (BD).

**Materials and methods:**

A cross-sectional study was conducted on 126 BD patients with current depressive episode (BDD), 102 BD patients with current mixed or (hypo)manic episode (BDM) and 94 healthy controls (HC). All participants were drug-naïve and had no current active physical illness associated with inflammatory response or history of substance abuse. Fasting serum levels of CRP, leptin (LEP), adiponectin (ADP), visfatin (VIS), TNF-α, IL-2, IL-6, IL-10, IL-17), and monocyte chemoattractant protein-1 (MCP-1) were measured with enzyme-linked immunosorbent assay (ELISA). Symptomatic severity of BD was assessed with HAMD-17 and YMRS. Generalized linear model was used to determine the association between the serum levels of inflammatory markers and symptomatic severity of BD.

**Results:**

The serum levels of IL-6, IL-10 and IL-17, and the IL-6/IL-10 ratio were significantly lower in mild BDD than in HC. In moderate BDD, the serum levels of MCP, IL-6 and IL-17 were significantly lower than in HC. In severe BDD, the serum level of ADP, MCP-1, IL-10 and IL-17and the IL-17/IL-10 ratio were significantly lower than in HC. The serum levels of TNF-α and the IL-6/IL-10 ratio were significantly higher in mild BDM than in HC. In moderate BDM, the serum level of VIS, IL-2, and IL-17 were significantly higher than in HC, but the IL-6/IL-10 ratio was significantly lower than in control. In severe BDM, the serum levels of IL-6 and IL-17 and the ratios of IL-6/IL-10 and IL-17/IL-10 were significantly lower than in HC, but the neutrophil/lymphocyte ratio was significantly higher than in HC.

**Conclusion:**

In BDD, immune-inhibition is persistently predominant, while in mild-to-moderate BDM, immune system is activated but inhibited in severe BDM. The dynamic change of serum inflammatory markers suggests that alteration of peripheral inflammatory markers in BD is state-dependent instead of trait-marked.

## Background

Over the past decade, growing and converging evidence has demonstrated that immune dysfunction is involved in the pathophysiology of BD (1). One of the most direct evident is the alteration of peripheral inflammatory markers is frequently reported in BD. However, the relationship between immune dysfunction and bipolar disorder has been far from clear, since results reported in individual studies related to the inflammatory system in BD have been inconsistent. Some studies (2, 3, 4) claim that the direction of alterations of cytokine levels in BD appears to be identical across affective states, indicating that the activation of the immune response system is a trait-marker of the disease. While other researches (5) find that elevated cytokines are seen in only acutely ill patients with BD, implying that immune system activation in BD is state-dependent and seen as a stress-related phenomenon. This inconsistency is thought to be attributed to heterogeneity between studies, insufficient standardization and lacking control for confounders (2), including gender (6), age (7), marital status (8), body mass index (BMI) (9), psychoactive substance abuse (6), atypical features (10), duration of illness (11), pharmaceutical treatment (12, 13), and so on. However, among all the potential confounding factors, symptomatic severity has been rarely purposefully studied.

Elevated pro-inflammatory cytokines had been reported to be associated with greater symptom burden in BD (14, 15), and correlation between severity of affective symptoms and serum levels of IL-6, sIL-2R, sIL-6R, TNF-α and tumor necrosis factor receptor 60 and 80 kDa (sTNFR60/80) was found in several studies (16–21). However, none of these positive findings was replicated. Beside lack of control of confounding factors, statistical method might be another reason for this non-repeatability. In the previous studies, the association between serum levels of inflammatory markers and symptomatic severity was treated as a linear relationship, which might simplify their actual connection. Supposing BD was an inflammatory disease as someone claimed (22), the serum levels of inflammatory markers would first increase with the worsening of symptoms and then decrease because of exhaustion with the evolvement of illness like other inflammatory diseases (23). In addition, among the peripheral inflammatory biomarkers, some are believed to be associated with mood states (state markers) while others are thought to be related to specific features of the long-term course of illness (trait markers) (24, 25). That is to say, the relationship between serum levels of inflammatory biomarkers and BD is far more complicated as supposed.

In this study, we are going to assess the relationship between serum levels of a wide variety of inflammatory cytokines and symptomatic severity in a large, drug-naive BD population in different illness episodes by controlling for as many confounding factors as possible. We sought to answer the following question: First, are there differences in serum levels of inflammatory markers among BD patients compared to healthy controls (HC)? Second, does different affective state affect serum levels of inflammatory markers differently? Finally, are there differences in serum levels of inflammatory markers among BD patients with varying severity of affective symptoms?

## Materials and methods

### Subjects

The sample consisted of 228 BD patients and 94 healthy controls. The cases came from the inpatients or outpatients who sought medical help in the psychiatric department of the Third Affiliated Hospital of Sun Yat-sen University between August 8, 2012 and January 6, 2018. The healthy controls were volunteers recruited from the local community during the same period. All the participants were Han Chinese, aged between 16 and 65, had no current active physical illness associated with inflammatory response, such as infectious and inflammatory diseases, carcinoma or tumor, and fresh injury of any kind, which confirmed by reviewing their previous medical records and routine clinical examination, had no history of psychoactive substance abuse in the past six months, and provided written informed consent. Participants who aged under 18 were required to provide written informed consent from their guardians. The cases had to meet the following criteria: (a) fulfill the diagnostic criteria of bipolar disorder of any kind according to the Diagnostic and Statistical Manual of Mental Disorders, Fourth Edition, Text Revision (DSM-IV-TR); (b) had not received any psychopharmaceutical treatment within 3 months prior to recruitment; (c) had no comorbid organic mental disorder. The HCs were screened for mental disorders using the Chinese version of the Structured Clinical Interview for the Diagnostic and Statistical Manual of Mental Disorders, Fourth Edition, Text Revision (DSM-IV-TR) Axis 1 Disorders (SCID-I), none patient version, and those with a current or history of major psychiatric disorders, dementia, mental retardation, would be excluded. In addition, participants who were pregnant or postpartum, under steroid or non-steroid anti-inflammatory drugs or other immune-inhibitors treatment, or too severely ill to cooperated with the required assessment were also excluded. This study was reviewed and approved by the Clinical Research Ethics Committee of the Third Affiliated Hospital of Sun Yat-sen University (approval number: [2017]02-233-01).

### Measurement

#### Diagnosis of BD

The diagnosis of BD was performed by board-certified psychiatrists (Zhaoyu Gan, Nianhong Guan, and Xiu hua Wu) according to the Diagnostic and Statistical Manual of Mental Disorders, Fourth Edition, Text Revision (DSM-IV-TR) criteria. The Chinese version of the Structured Clinical Interview for DSM- IV-TR Axis 1 Disorders (SCID-I), patient version was used as an auxiliary tool for performing diagnosis.

#### Assessment of clinical characteristics of BD

General demographic information of the participants was collected through a self-designed questionnaire. Psychotic features were measured by evaluating whether the participants experienced any psychotic symptoms including hallucination, delusion or disorganized behavior, in the past affective episodes. Physical comorbidities were confirmed by reviewing the patients’ previous medical history and medical records stored in our hospital’s electronic medical system. Diagnoses of mental comorbidities were made according to the DSM-IV-TR based on the subjects’ history of present illness and routine mental examination. Family history of mental disorders was assessed by asking the subjects or their accompanying relatives whether their first or second-degree relatives had mental disorders of any kind. All the above-mentioned interviews were conducted by trained psychiatrists in our study team.

#### Measurement of the symptomatic severity of BD

The symptomatic severity of BD was evaluated with the 17-item Hamilton Depression Scale (HAMD-17) (26) and the Young Mania Rating Scale (YMRS) (27). To make sure the quality and reliability of the assessment, it was performed by the same well-trained researcher (Zhaoyu Gan) on all the cases. The scores of HAMD-17 and YMRS were divided into four levels, for HAMD-17 (28): 0 (no depression): ≤ 7; 1(mild depression): >7 and <17;2 (moderate depression): ≥ 17 and < 24; 3 (severe depression): ≥ 24; for YMRS: 0 (no mania): < 6; 1 (mild mania): ≥ 6 and < 13; 2 (moderate mania): ≥ 13 and < 19; 3 (server mania): ≥ 19.

#### Biochemical measurement

Ten milliliters of fasting blood were withdrawn between 7:00 am and 9:00 am from each subject by venipuncture into two free-anticoagulant vacuum tubes. One tube of blood was immediately sent to the library of our hospital for routine blood test and measurement of C reactive protein (CRP). Routine blood test was performed in an automated blood cell counter (Hiesen Mikang Co., Ltd., Kőbe, Japan). CRP was measured by immune transmission turbidimetry with a biochemical analyzer (Sichuan Mike Biological Co., Ltd., and Nippon ihua Co., Ltd., Tokyo, Japan). The other tube of blood was immediately centrifuged at 3,000 g for 5 min, and the serum was kept frozen at −80°C until assayed. The concentrations of leptin (LEP), adiponectin (ADP), visfatin (VIS), tumor necrosis factor-α (TNF-α), interleukin-2 (IL-2), interleukin-6 (IL-6), interleukin-10 (IL-10), interleukin-17 (IL-17), and monocyte chemoattractant protein-1 (MCP-1) were assessed with enzyme-linked immunosorbent assay (ELISA) using assay from Shanghai Caiyou Industrial Co., Ltd. (The product number, sensitivity, intra- and inter-assay coefficients, and relativity of the above-mentioned ELISA kits were listed in Table 1.

**TABLE 1:** The product number, sensitivity, intra- and inter-assay coefficients, and relativity of the ELISA kits.

ELISA kit	Product number	Sensitivity	Inter-assay coefficients	Intra-assay coefficients	Relativity
LEP	YS01714B	0.075 μg/L	9.1%	7.2%	0.9998
ADP	YS05573B	20 pg/ml	9.1%	7.2%	0.9998
VIS	YS02016B	3.75 pg/ml	9.1%	7.2%	0.9998
TNF-α	YS04866B	5 ng/L	9.1%	7.2%	0.9998
IL-2	YS04838B	10 pg/ml	9.1%	7.2%	0.9998
IL-6	YS04835B	0.2 ng/L	9.1%	7.2%	0.9998
IL-10	YS04842B	2.5 ng/L	9.1%	7.2%	0.9998
IL-17	YS01053B	0.175 ng/L	9.1%	7.2%	0.9998
MCP-1	YS06680B	2.5 ng/L	9.1%	7.2%	0.9998
CRP	926RGS	0.1 mg/L	5%	10%	0.990

LEP, leptin; ADP, adiponectin; VIS, visfatin; TNF-α, tumor necrosis factor-α; IL-2, interleukin-2; IL-6, interleukin-6; IL-10, interleukin-10; IL-17, interleukin-17; MCP-1, monocyte chemoattractant protein-1.

#### Statistical analysis

Participants were categorized into BD group and HC group according to the inclusion criteria, and the BD group was further divided into two subgroups——depressive group and (hypo)manic or mixed group based on their current episode. Patients with current mixed episode was classified as (hypo) manic group, considering the number of patients with current (hypo)manic episode was small in this study and (hypo)manic and mixed episode shared similar treatment strategy (29). For normally distributed data, independent-sample *t*-test was used to test the difference between groups; while for non-normally distributed variable, comparison between groups was analyzed with Wilcoxon rank sum test. Difference in categorical parameters between groups was tested using Chi-square test. Considering serum levels of inflammatory markers were not normally distributed and their relationship with severity of BD was not linear, generalized linear model was chosen (30) to assess their relationship. Odds ratios (OR) and 95% confidence intervals (95% CI) were used to quantify the strength of associations. The Bonferroni adjusted significance test was used for multiple comparisons. All data were analyzed using commercial statistical package SPSS 24.0 (SPSS, Inc., Chicago, IL).

## Results

### Demographic and clinical characteristics of participants

As seen in Table 2, 126 BD patients with current depressive episode (BDD), 102 BD patients with current (hypo)manic or mixed episode (BDM) and 94 HCs were recruited in this study. The level of education and the proportion of female participants among BDD were significantly lower than those among HC (*P* < 0.001), but no significant difference was found in age, marital status, and BMI between BDD and HC. Compared to HC, BDM was younger (*P* < 0.001) and had a lower level of education (*P* < 0.001), but did not significantly differ in gender proportion, marital status and BMI.

**TABLE 2:** Demographic and clinical characteristics of all the participants.

	Bipolar disorder		Control *N* = 94	*P* ^1^	*P* ^2^
	Depressive *N* = 126	(hypo)manic *N* = 102			
Age (mean ± SD)(year)	27.8 ± 10.7	25.4 ± 8.1	29.6 ± 11.0	0.241	**0**.**002**
Female *n*(%)	65(51.6)	66 (64.7)	68(72.3)	**0**.**002**	0.284
Education (mean ± SD)(year)	13.0 ± 3.4	12.8 ± 3.0	16.2 ± 3,5	**<0**.**001**	**<0**.**001**
Married *n* (%)	42(33.3)	29(28.4))	28(29.8)	0.661	0.876
BMI (mean ± SD)	21.7 ± 2.9	21.5 ± 3.6	21.5 ± 3.8	0.674	0.997
Onset age (mean ± SD)(year)	23.5 ± 9.3	21.1 ± 8.6			
Duration of illnes course (min-max, median) (year)	0.04-38, 2.0	0.02-33, 2.0			
**Severity of symptoms**					
1 (mild)	17	22	NA		
2 (moderate)	35	37	NA		
3 (severe)	74	43	NA		

^1^Bipolar depression compared with Control.

^2^Bipolar disorder with current (hypo)manic or mixed episode compared with Control.

^3^BMI: body mass index.

^4^Bold font denotes *p* < 0.05.

Table 3 compared the clinical characteristics of BD of different severities. As a result, no significant difference was found in physical or mental comorbidities (totally or any specific subgroup), duration of illness course, psychotic features and family history of mental disorders among BDD or BDM of different severities (all *P* > 0.05).

**TABLE 3:** Comparison of clinical characteristics among BD of different severities.

	Depressive			*P*	(hypo)manic			*P*
	Mild (*N* = 17)	Moderate (*N* = 35)	Severity (*N* = 74)		Mild (*N* = 22)	Moderate (*N* = 37)	Severity (*N* = 43)	
Physical co-morbidities	3	6	19	0.796	5	5	20	0.170
Inflammatory or Autoimmune disease^a^	1	3	5	0.921	1	0	3	0.273
Cyst or hyperplasia^b^	1	1	7	0.447	1	4	9	0.156
Thyroid diseases^c^	0	0	2	0.490	1	1	1	0.877
Glycolipid metabolic diseases^d^	1	1	3	0.870	1	0	4	0.157
Cardiovascular diseases^e^	0	1	2	0.786	1	0	3	0.273
Mental comorbidities	2	9	24	0.445	6	13	15	0.884
OCD	2	4	5	0.645	1	3	4	0.794
Anxiety disorders^f^	0	2	2	0.510	1	2	3	0.914
Eating disorders^g^	0	1	1	0.718	1	4	1	0.262
Somatoform disorders	0	1	7	0.215	2	1	2	0.554
Attempted suicide^h^	0	0	2	0.490	0	0	1	0.500
Duration of illness course				0.166				0.104
≤1 year	3	12	23		4	14	10	
>1 year and ≤ 3 years	9	16	22		11	10	13	
>3 years	5	7	27		4	13	19	
Psychotic features	3	4	13	0.699	6	10	14	0.83
Family history of mental disorders	2	7	10	0.622	4	11	9	0.522

^a^Including all kinds of asymptomatic infectious diseases, allergic rhinitis, urticaria, eczema, psora, asthma, and irritable bowel syndrome.

^b^Including all the benign cyst, nodule, polyp and well-healed cancer.

^c^Including Grave’s diseases, thyroiditis, thyroid cyst, thyroid nodule, thyroid cancer., primary hypothyroidism.

^d^Including diabetes mellitus, impaired glucose tolerance, and hepatic adipose infiltration.

^e^Including hypertension, lacunar cerebral infarction, aneurism, phlebangioma, atherosclerosis, and varicosity.

^f^Including panic disorder, generalized anxiety disorder, and social phobia.

^g^Including bulimia nervosa and anorexia nervosa.

^h^Failed suicide attempt in the last two weeks.

### The association between severity of depressive symptoms and serum level of inflammatory markers

Table 4 listed the detailed result about the serum levels of inflammatory markers in each group. Table 5 further demonstrated that compared to HC, most of the observed inflammatory markers, including pro-inflammatory and anti-inflammatory markers, showed a trend of decrease in BDD of any degree. In mild BDD, the serum level of IL-6(*P* = 0.003, OR = e^–1.087^ = 0.337, 95%CI = e^–1.807^-e^–0.366^ = 0.164-0.693), IL-10(*P* = 0.002, OR = e^–1.069^ = 0.343, 95%CI = e^–1.746^-e^–0.393^ = 0.174-0.675) and IL-17(*P* < 0.001, OR = e^–1.203^ = 0.300, 95%CI = e^–1.81^-e^–0.595^ = 0.164-0.551) were significantly lower than those in HC, the IL-6/IL-10 ratio was significantly lower (*P* = 0.008, OR = e^–0.892^ = 0.410, 95%CI = e^–1.544^-e^–0.23^ = 0.311-0.990) in mild BDD than in health control, but after Bonferroni correction, the difference did not reach significance. In moderate BDD, the serum level of MCP (*P* = 0.002, OR = e^–733^ = 0.480, 95%CI = e^–1.194^-e^–0.272^ = 0.303-0.762), IL-6 (*P* < 0.001, OR = e^–0.928^ = 0.395, 95%CI = e^–1.41^-e^–0.446^ = 0.244-0.640) and IL-17 (*P* < 0.001, OR = e^–1.035^ = 0.355, 95%CI = e^–1.478^-e^–0.593^ = 0.228-0.553) were significantly lower than those in HC, but no significant difference was found in the ratios of IL-2/IL-10, IL-6/IL-10, IL-17/IL-10 and neutrophil/lymphocyte (N/L) between BDD and HC. In severe BDD, the serum level of ADP (*P* = 0.002, OR = e^–0.81^ = 0.445, 95%CI = e^–0.915^-e^–0.199^ = 0.400-0.820), MCP-1(*P* < 0.001, OR = e^–0.557^ = 0.573, 95%CI = e^–1.179^-e^–0.44^ = 0.308-0.644), IL-10(*P* = 0.001, OR = e^–0.616^ = 0.540, 95%CI = e^–0.979^-e^–0.253^ = 0.376-0.776, and IL-17(*P* < 0.001, OR = e^–1.235^ = 0.291, 95%CI = e^–1.607^-e^–0.863^ = 0.200-0.422) were significantly lower than those in HC, and the IL-17/IL-10 ratio was also lower (*P* < 0.001, OR = e^–0.752^ = 0.471, 95%CI = e^–1.091^-e^–0.412^ = 0.335-0.662).

**TABLE 4:** The serum levels of inflammatory markers of bipolar disorder of different severities.

Inflammatory markers	BPD			BPM			Control (*N* = 94)
	Mild (*N* = 17)	Moderate (*N* = 35)	Severe (*N* = 74)	Mild (*N* = 22)	Moderate (*N* = 37)	Severe (*N* = 43)	
WBC (mean ± SD) (10e9/L)	5.90 ± 0.91	6.34 ± 1.53	6.70 ± 1.96	6.76 ± 1.58	7.06 ± 2.04	6.53 ± 2.07	6.30 ± 1.89
CRP (mean ± SD) (g/l)	0.98 ± 1.09	0.93 ± 0.92	0.84 ± 0.91	0.87 ± 0.63	1.77 ± 4.76	1.30 ± 2.20	1.10 ± 1.59
ADP (min-max, median) (pg/ml)	483.0-7945, 2735	117.0-8175.0, 2448.0	142.0-30600.0, 2265.0	133.0-117200.0, 3324.0	415.0-95830.0, 2382.0	402.0-52600.0, 2160.0	17.0-13520.0, 1320.0
LEP (min-max, median) (μg/L)	6.3-60.0, 22.5	0.88-563.0,19.8	0.31-296.1, 14.2	2.0-454.0, 17.5	3.5-768.8, 20.6	3.5-157.0, 14.9	0.8-729.7, 8.2
VIS (min-max, median) (pg/ml)	98-1666.0, 241.0	15.2-1927.0, 393.0	11.4-9790.0, 302.0	98.0-3950.0, 423.0	108.0-29900.0, 390.0	75.8-5898.0, 396.0	4.0-12550.0, 205.0
MCP-1 (min-max, median) (ng/L)	48.0-745.0,256.0	4.50-1200.0, 240.0	9.7-5600.0, 188.0	1.0-2430.0, 4114.0	39.0-11530.0, 313.0	35.0-3734.0, 212.0	0.8-18530.0, 240.0
IL-2 (min-max, median) (pg/ml)	214.0-2695.0, 914.0	225.0-2449.0, 797.0	114.0-5329.0, 980.0	350.0-8915.0, 1571.0	207.0-40940.0, 950.0	154.0-5810.0, 894.5	93.0-7500.0, 435.0
IL-6 (min-max, median) (ng/L)	4.80-75.5, 12.8	0.60-85.0, 21.4	0.37-796.0, 20.6	4.8-280.0, 20.5	5.4-1250.0, 27.0	4.2-337.2, 21.0	0.5-1128.0, 20.0
IL-10 (min-max, median) (ng/L)	180.0-929.5, 358.0	0.80-3045.0, 573.0	0.80-8730.0, 458.0	0.80-3920.0, 532.0	191.0-20310.0, 683.0	67.7-19010.0, 497.3	0.7-20960.0, 511.0
IL-17 (min-max, median) (ng/L)	6.2-59.0, 20.0	4.3-65.0, 26.0	2.6-75.0, 14.6	10.5-377.6, 24.8	2.9-4883.0, 21.5	2.4-96.0, 18.6	5.5-813.0, 20.0
TNF-α (min-max, median) (ng/L)	134.0-2695.0, 358	10.0-1600.0, 460.0	2.0-8995.0, 362.0	5.0-20630.0, 569.0	120.0-8086.0, 693.0	100.0-8542.0, 412.0	6.0-8021.0, 390.0
N/L (min-max, median)	0.68-2.16, 1.3	0.55-4.60, 1.54	0.61-4.03, 1.49	0.97-3.83, 1,78	0.74-10.82, 1.48	0.26-54.22, 1.41	0.47-6.39, 1.49
IL-2/IL-10 (min-max, median)	0.62-9.56, 1.57	0.34-8.94, 1.40	0.38-11.91, 2.01	0.39-7.39, 3.03	0.14-21.81, 1.44	0.26-4.81, 1.66	0.07-17.06, 0.97
IL-6/IL-10 (min-max, median)	0.01-0.15, 0.05	0.00-0.75, 0.04	0.00-1.00, 0.47	0.01-6.25, 0.05	0.00-0.67, 0.04	0.01-0.20, 0.04	0.00-1.71, 0.04
IL-17/IL-10 (min-max, median)	0.01-0.16,0.05	0.01-0.61, 0.03	0.01-0.13, 0.03	0.02-0.48, 0.05	0.01-2.30, 0.04	0.00-0.23, 0.03	0.00-1.01, 0.04

**TABLE 5:** The association between severity of depressive symptoms and serum level of inflammatory markers.

Inflammatory markers	*N*	Goodness of fit (value/df)	Omnibus Test (*P*值)	BDD^1^											
				Mild				Moderate				Severe			
				β^2^	95%CI		*P* ^3^	β^2^	95%CI		*P* ^3^	β^2^	95%CI		*P* ^3^
WBC^4^	156	3.312	0.463	−0.409	−1.606	0.789	0.504	0.037	−0.793	0.868	0.930	0.400	−0.241	1.042	0.221
CRP^5^	172	0.951	0.535	−0.121	−0.691	0.449	0.677	−0.175	−0.593	0.243	0.412	−0.225	−0.535	0.085	0.155
ADP^5^	210	1.612	0.013	−0.482	−1.149	0.185	0.157	−0.481	−0.928	−0.034	0.035	−0.557	−0.915	−0.199	**0.002**
LEP^5^	210	1.591	0.113	−0.295	−0.0959	0.368	0.383	0.188	−0.257	0.632	0.407	−0.330	−0.686	0.026	0.069
VIS^5^	210	1.322	0.512	−0.255	−0.866	0.357	0.414	−0.218	−0.627	0.191	0.297	−0.217	−0.545	0.111	0.196
MCP-1^5^	210	1.730	<0.001	−0.904	−1.592	−0.215	0.010	−0.733	−1.194	−0.272	**0.002**	−0.810	−1.179	−0.440	**< 0.001**
IL-2^5^	156	0.794	0.392	−0.048	−0.552	0.457	0.853	−0.162	−0.530	0.205	0.386	0.153	−0.156	0.462	0.331
IL-6^5^	210	1.914	<0.001	−1.087	−1.807	−0.366	**0.003**	−0.928	−1.410	−0.446	**<0.001**	−0.544	−0.930	−0.158	0.006
IL-10^5^	210	1.665	0.001	−1.069	−1.746	−0.393	**0.002**	−0.581	−1.034	−0.128	0.012	−0.616	−0.979	−0.253	**0.001**
IL-17^5^	156	1.197	<0.001	−1.203	−1.810	−0.595	**<0.001**	−1.035	−1, 0.478	−0.593	**<0.001**	−1.235	−1.607	−0.863	**< 0.001**
TNF-α^5^	210	1.215	0.405	−0.106	−0.694	0.483	0.725	−0.319	−0.714	0.075	0.112	0.027	−0.288	0.343	0.865
N/L^6^	154	0.206	0.328	−0.182	−0.476	0.113	0.226	0.003	−0.201	0.208	0.975	0.087	−0.071	0.246	0.280
IL-2/IL-10^5^	156	0.921	0.403	0.382	−0.157	0.922	0.165	−0.024	−0.417	0.369	0.904	0.162	−0.169	0.493	0.337
IL-6/IL-10^5^	210	1.584	0.032	−0.892	−1.554	−0.230	0.008	−0.361	−0.805	0.082	0.110	−0.368	−0.724	−0.013	0.042
IL-17/IL-10^5^	156	0.978	0.001	−0.319	−0.874	0.235	0.257	−0.296	−0.699	0.108	0.151	−0.752	−1.091	−0.412	**< 0.001**

^1^Adjustment for age, gender, marital status, BMI, education, and severity of manic symptoms.

^2^Taking the healthy control group as reference, if the β is positive, the level of inflammatory marker is higher than that of healthy control group, and if the β is negative, the level of inflammatory marker is lower than that of healthy control group.

^3^Bold font denotes *p* < 0.05/15 = 0.0033.

^4^According to the scatter plot and statistical test, these data are approximately normally distributed, so the linear model is used in modeling.

^5^According to the scatter plot and statistical test, these data are skewed distributed, but approximately normally distributed after log conversion, so gamma with log link model is chosen for modeling.

^6^N/L: Granulocyte/lymphocyte ratio.

### The association between severity of manic symptoms and serum level of inflammatory markers

Table 6 showed a different change model of serum level of inflammatory markers across manic symptoms of varying degree. In mild BDM, only the serum level of TNF-α (*P* < 0.001, OR = e^1.000^ = 2.718, 95%CI = e^0.490^-e^1.511^ = 1.632-4.531) was significantly higher than that in HC, but the IL-6/IL-10 ratio was significantly lower (*P* < 0.001, OR = e^1.141^ = 3.130, 95%CI = e^0.586^-e^1.696^ = 1.797-5.452) in case than in control. In moderate BDM, the serum levels of VIS (*P* < 0.001, OR = e^0.862^ = 2.368, 95%CI = e^0.419^-e^1.305^ = 1.520-3.688), IL-2(*P* < 0.001, OR = e^0.983^ = 2.672, 95%CI = e^0.606^-e^1.361^ = 1.833-3.900), and IL-17 (*P* < 0.001, OR = e^1.375^ = 3.955, 95%CI = e^0.951^-e^1.798^ = 2.588-6.038), were seen to be significantly higher than in HC, but the IL-6/IL-10 ratio was also significantly lower (*P* = 0.003, OR = e^–0.685^ = 0.504, 95%CI = e^–1.141^-e^–0230^ = 0.319-0.794) in the case group. However, in severe BDM, most of the observed pro-inflammatory markers were lower in the case group than in HC. Of them, IL-6 (*P* = 0.001, OR = e^–0.813^ = 0.443, 95%CI = e^–1.281^-e^–0.346^ = 0.278-0.708) and IL-17 (*P* < 0.001, OR = e^–1.110^ = 0.330, 95%CI = e^–1.716^-e^–0.504^ = 0.180-0.604) reached significance. In addition, the ratios of IL-6/IL-10 (*P* < 0.001, OR = e^–1.011^ = 0.364, 95%CI = e^–1.442^-e^–0.580^ = 0.236-0.560) and IL-17/IL-10 (*P* = 0.001, OR = e^–0.761^ = 0.467, 95%CI = e^–1.209^-e^–0.314^ = 0.298-0.730) were significantly lower in the case group than in HC. On contrary, the N/L ratio was found to be significantly higher in severe BDM than in HC (P = 0.001, OR = e^0.499^ = 1.647, 95%CI = e^0.212^-e^0.787^ = 1.236-2.198). The detailed result regarding to the serum levels of inflammatory markers in each group was listed in Table 4.

**TABLE 6:** The association between severity of manic symptoms and serum level of inflammatory markers.

Inflammatory markers	N	Goodness of fit (value/df)	Omnibus Test (*P*值)	BDM^1^											
				Mild				Moderate				Severe			
				β^2^	95%CI		*P* ^3^	β^2^	95%CI		*P* ^3^	β^2^	95%CI		*P* ^3^
WBC^4^	146^1^	3.807	0.345	0.462	−0.685	1.609	0.430	0.761	−0.074	1.595	0.074	0.228	−0.538	0.994	0.560
CRP^5^	161^6^	1.452	0.065	−0.238	−0.800	0.323	0.406	0.472	0.074	0.869	0.020	0.166	−0.199	0.531	0.374
ADP^5^	189	2.196	0.022	0.604	−0.018	1.227	0.057	0.401	−0.116	0.918	0.128	−0.319	−0.802	0.164	0.196
LEP^5^	190	1.6741	0.010	0.309	−0.244	0.862	0.273	0.458	0.004	0.912	0.048	−0.412	−0.814	0.017	0.060
VIS^5^	190	1.584	<0.001	0.068	−0.472	0.608	0.805	0.862	0.419	1.305	**<0.001**	−0.281	−0.700	0.138	0.189
MCP-1^5^	191	2.029	0.082	−0.355	−0.957	0.246	0.247	0.023	−0.471	0.517	0.929	−0.584	−1.047	−0.121	0.014
IL-2^6^	144	52.166	<0.001	0.499	0.004	0.995	0.048	0.983	0.606	1.361	**<0.001**	0.134	−0.286	0.554	0.532
IL-6^5^	190	2.035	0.007	−0.355	−0.958	0.248	0.248	0.106	−0.388	0.601	0.674	−0.813	−1.281	−0.346	**0.001**
IL-10^5^	191	1.854	0.22	−0.588	−1.167	−0.010	0.046	0.086	−0.389	0.561	0.723	−0.177	−0.622	0.268	0.436
IL-17^6^	144	26.162	<0.001	−0.395	−1.083	0.293	0.260	1.375	0.951	1.798	**<0.001**	−1.110*	−1.716	−0.504	**< 0.001**
TNF-α^6^	190	1.396	NA^7^	1.000	0.490	1.511	**<0.001**	0.440	0.021	0.860	0.039	0.014	−0.382	0.410	0.944
N/L^8^^,^^5^	144	0.573	0.009	0.163	−0.266	0.592	0.456	0.217	−0.095	0.5303	0.173	0.499	0.212	0.787	**0.001**
IL-2/IL-10^5^	144	0.928	0.077	0.450	−0.027	0.926	0.064	0.219	−0.161	0.598	0.259	−0.150	−0.526	−0.226	0.434
IL-6/IL-10^5^	190	1.687	<0.001	1.141	0.586	1.696	**<0.001**	−0.685	−1.141	−0.230	**0.003**	−1.011	−1.442	−0.580	**< 0.001**
IL-17/IL-10^5^	144	1.368	0.001	−0.028	−0.595	0.539	0.922	0.318	−0.133	0.769	0.167	−0.761	−1.209	−0.314	**0.001**

^1^Adjustment for age, gender, marital status, BMI, education, and severity of depressive symptoms.

^2^Taking the healthy control group as reference, if the β is positive, the level of inflammatory marker is higher than that of healthy control group, and if the β is negative, the level of inflammatory marker is lower than that of healthy control group.

^3^Bold font denotes *p* < 0.05/15 = 0.0033.

^4^According to the scatter plot and statistical test, these data are approximately normally distributed, so the linear model is used in modeling.

^5^According to the scatter plot and statistical test, these data are skewed distributed, but approximately normally distributed after log conversion, so gamma with log link model is chosen for modeling.

^6^The data dispersion is too high to be modeled when linear or gamma with log link mode is used, so tweedie with log link mode is used instead.

^7^Unable to compute the initial model log likelihood due to numerical problems.

^8^N/L: Granulocyte/lymphocyte ratio.

## Discussion

We investigated the relationship between serum levels of inflammatory markers and symptomatic severity of bipolar disorder among 228 drug-naïve patients with bipolar disorder. Our study has three major findings. First, in BDD, both pro-inflammatory markers and anti-inflammatory markers are lower than those in HC in spite of the symptomatic severity. Second, in BDM, the serum levels of pro-inflammatory markers elevate in mild-to-moderate stage and decrease in severe one. Third, the imbalance of pro-inflammatory markers and anti-inflammatory markers is seen in both BDD and BDM, which is shown by an alteration (decrease in BDD and increase in BDM) in the IL-6/IL-10 ratio when the symptoms are mild and a decrease in the IL-17/IL-10 ratio when the symptoms are severe.

Contrary to the view from studies in major depressive episode (MDD) (31, 32) that serum levels of both pro-inflammatory cytokines and ant-inflammatory cytokines slightly increase in depressive episode, our study finds that both pro-inflammatory cytokines and anti-inflammatory cytokines decrease in bipolar depression. In addition, the decrease of the ratios of IL-6/IL-10 and IL-17/IL-10 found in this study also suggests that immune-inhibition is prominent in bipolar depression, especially in severe BD. Different from studies in MDD, few studies have ever assessed the relationship between the serum levels of inflammatory markers and depressive symptoms from studies in BDD. Several studies with small sample size have found that bipolar depression is associated with a pro-inflammatory state (33, 34). However, in two review studies (2, 35) which meta-analyzed the cytokines alteration in BD, no consistent conclusion about the relationship between cytokines and bipolar depression was reached since few studies on bipolar depression were included and the sample sizes of the included studies were all small. In addition, the heterogeneity between studies also played a part in this inconsistency (35). A recent comparison study (36) base on a machine learning approach found that compared to HC, BDD had higher levels of C-C Motif Chemokine Ligand (CCL)3, CCL4, CCL5, CCL11, CCL25, CCL27, CXCL11, IL-9, and TNF-α. However, in this study, all the participants were medicated, potential confounders including age, gender, BMI, and tobacco or alcohol use were not controlled. In addition, the average age of the participants were bigger than those in our study (46.59 ± 10.8 VS. 27.8 ± 10.7), which might be another factor that makes their conclusion different from ours. In addition, the foregoing conclusion could not be duplicated by other studies either. For example, a study (37) from German showed that both pro-inflammatory and anti-inflammatory markers, but not CRP were inversely correlated with the severity and symptoms of major depression. A recent study (38) from China found that the serum levels of IL-13 and TNF-α were significantly lower in BDD than in MDD, and the serum levels of IL-4 and TNF-α increased in the treatment response subgroup of BDD. In recent years, anti-inflammatory agents were expected to be a promising treatment target of MDD (39) and BDD (40). However, negative results from large clinical trials (41, 42) not only overturns the conclusion from small studies that the adjunctive use of anti-inflammatory drugs might help improve depressive symptoms in MDD or BDD, but also make us rethink the relationship between serum levels of inflammatory cytokines and depressive symptoms. Moreover, pro-inflammatory cytokines are not always neurotoxic. Instead, low-dose of pro-inflammatory cytokines such as IL-6, TNF-a, and IFN-γ have been proved to have a neuroprotective role (43).

The relationship between serum levels of inflammatory markers and BDM has been widely studied (44). Most of previous studies find that both pro-inflammatory cytokines (IL-1RA (45), IL-1, Il-2 (46), sIL-2R (47, 48), IL-6 (46, 49), sIL-6R (47), TNF-α (49), CXCL10 (50), CXCL11 (50), CRP (47), IL-17 (51), IFN-γ (34)and IL-18 (52)) and anti-inflammatory markers (IL-4 (46) and IL-10 (53)) elevate in the manic episode. However, our study only partially supports this view. In our study, the elevation of pro-inflammatory markers was seen only in mild and moderate BDM, while in severe BDM the serum levels of pro-inflammatory were found to decrease. This finding is consistent with our previous hypothesis about the dynamic change of inflammatory markers over the course of a multi-system inflammatory disease. As far as we know, this has rarely been explored before. Although serum levels of some inflammatory cytokines like sIL-2R (48), IL-17 (51), neural cell adhesion molecule 1(NCAM-1) (53), carcinoembryonic antigen (CEA) (54) and IFN-γ (34) have been reported to be positively correlated with severity of manic symptoms, the relationship between serum levels of inflammatory markers, according to our study, seems to be more complicated than a positive correlation.

Inflammatory ratios except N/L, which are less affected by exercise, BMI, and other confounding factors than other commonly used markers of inflammation (55), have been rarely studied in BD before. Partially in line with previous reports (56) that N/L ratio is higher in BDM than in BDD or MDD, our study finds that N/L ratio is significantly higher in severe BDM than in HC, suggesting an imbalance in favor of innate immunity (57). In addition, we also find that the ratios of IL-6/IL-10 and IL-17/IL-10 vary with the clinical phase of BD and severity of symptoms: in BDD, the IL-6/IL-10 ratio decrease in mild BDD and the IL-17/IL-10 ratio decrease in severe BDD; while in BDM, the IL-6/IL-10 ratio elevate in mild BDM and decrease with the IL-17/IL-10 ratio in severe BDM. That is to say, the IL-6/IL-10 imbalance is an early immune response to BD, while IL-17/IL-10 imbalance is an indicator of deterioration of BD. To our knowledge, these have not been reported before. Although the pathophysiological meaning of IL-6/IL-10 and IL-17/IL-10 balance is far from being interpreted clearly, it is possible to suppose that they play a role in homing of inflammatory cells and therefore in the outcome of inflammation (58).

This study has several strengths. First of all, it comprises of one of the largest sample of BD patients ever examined. Second, all the subjects were Han Chinese and drug-naïve, most of them were young, and active physical diseases and recent substance abuse were excluded, which provided us a good sample with great homogeneity. Third, generalized linear model was used to statistically analyze the data, whereas as many potential confounding factors as possible were adjusted for, so the power of test greatly improved. Fourth, as many inflammatory cytokines as possible were measured at the same time, which makes the assessment of the immune state of the subjects more multidimensional. Finally, all the blood samples were treated in the same way and in the same laboratory, thus minimized the effect of measurement deviation. However, several limitations should be addressed when interpreting the foregoing conclusion. First, its cross-sectional design limits causal conclusion about the relationship between serum levels of inflammatory markers and BD. Second, althougth some potential confounders like duration of illness course, psychotic features and comorbidities were balanced among BD of different severities, other factors like cognitive symptoms and atypical features were not concerned, which had been proved to be associated with the levels of inflammation. Third, although we have the largest sample of BD ever examined, the sample might be still not big enough to detect some small but clinically meaningful effect of inflammatory marker on the symptomatic severity of BD, especially when the sample is divided into several subgroups. Fourth, some but fortunately not much inflammatory markers’ data was too dispersed to build a good generalized linear model, therefore caution should be exercised when interpreting the corresponding results. Fifth, no patient in remission was enrolled in this study, so the conclusion drawn from this study could not reflect the full picture of dynamic change over the severity of illness. Finally, we only measured the serum levels of inflammatory markers instead of those in central nervous system. Therefore, we did not know whether the inflammatory markers in the central nervous system in BD might follow the similar dynamic change model with the varying of symptomatic severity.

## Conclusion

The serum level of inflammatory markers and the balance between pro-inflammatory markers and anti-inflammatory markers not only vary with the types of affective episode but also with the severity of affective symptoms. In BDD, immune-inhibition is persistent predominant, while in BDM, the dysfunction of immune system varies with severity of affective symptoms: in mild-to-moderate BDM, the immune system is activated while inhibited in severe BDM. The dynamic change of serum inflammatory markers suggests that alteration of peripheral inflammatory markers in BD is state-dependent instead of trait-marked.

## Data availability statement

The original contributions presented in this study are included in this article/Supplementary material, further inquiries can be directed to the corresponding authors.

## Ethics statement

The studies involving human participants were reviewed and approved by the Clinical Research Ethics Committee of the Third Affiliated Hospital of Sun Yat-sen University. Written informed consent to participate in this study was provided by the participants’ legal guardian/next of kin.

## Author contributions

ZG and NG conceived and designed the study and revised it for intellectual content. ZG, XW, YL, ZY, and NG took recruitment and management of the case. ZC was in charge of biochemical measurement. XW, ZC, and XL contributed to data analysis and interpretation. XW and ZC drafted the manuscript. All authors read and approved the final manuscript.
